# Accelerated Deficits of Spatial Learning and Memory Resulting From Prenatal Inflammatory Insult Are Correlated With Abnormal Phosphorylation and Methylation of Histone 3 in CD-1 Mice

**DOI:** 10.3389/fnagi.2019.00114

**Published:** 2019-05-16

**Authors:** Zi-Xing Wu, Lei Cao, Xue-Wei Li, Wei Jiang, Xue-Yan Li, Jing Xu, Fang Wang, Gui-Hai Chen

**Affiliations:** ^1^Department of Neurology, The First Affiliated Hospital of Anhui Medical University, Hefei, China; ^2^Department of Neurology, Nanjing Drum Tower Hospital, Nanjing, China; ^3^Department of Neurology, The Second Affiliated Hospital of Anhui Medical University, Hefei, China; ^4^Department of Neurology, The First Affiliated Hospital of University of South China, Hengyang, China; ^5^Departments of Neurology and Sleep Disorders, The Affiliated Chaohu Hospital of Anhui Medical University, Hefei, China

**Keywords:** aging, histone, lipopolysaccharide, memory, methylation, phosphorylation

## Abstract

Gestational infection causes various neurological deficits in offspring, such as age-related spatial learning and memory (SLM) decline. How inflammation causes age-related SLM dysfunction remains unknown. Previous research has indicated that histone modifications, such as phosphorylation of H3S10 (H3S10p) and trimethylation of H3K9 (H3K9me3) may be involved. In our study, pregnant mice received an intraperitoneal injection of lipopolysaccharide (LPS, 50 or 25 μg/kg) or normal saline during gestational days 15–17. After normal parturition, the offspring were randomly separated into 1-, 6-, 12-, 18-, and 22-month-old groups. SLM performance was assessed using a radial six-arm water maze (RAWM). The hippocampal levels of H3S10p and H3K9me3 were detected using an immunohistochemical method. The results indicated that the offspring had significantly impaired SLM, with decreased H3S10p and increased H3K9me3 levels from 12 months onward. Maternal LPS exposure during late gestation significantly and dose-dependently exacerbated the age-related impairment of SLM, with the decrease in H3S10p and increase in H3K9me3 beginning at 12 months in the offspring. The histone modifications (H3S10p and H3K9me3) were significantly correlated with impairment of SLM. Our findings suggest that prenatal exposure to inflammation could exacerbate age-related impairments of SLM and changes in histone modifications in CD-1 mice from 12 months onward, and SLM impairment might be linked to decreased H3S10p and increased H3K9me3.

## Introduction

Cognitive abilities, such as learning and memory, commonly decline with age. However, the mechanism underlying age-associated memory impairment remains unknown. According to the hypothesis of “fetal origins of adult disease” (Osmond and Barker, 2000; Calkins and Devaskar, 2011), prenatal disturbances are associated with psychiatric and neurological disorders later in life (Batinic et al., 2016).

Maternal bacterial infections, which cause systemic inflammation, are the most common adverse exposure during the human fetal period (Khan et al., 2017). In mammals, peripheral inflammation induced by lipopolysaccharide (LPS) has been confirmed to be able to induce poor neurobehavioral performance in the elderly mammals (Bakos et al., 2004; Izvolskaia et al., 2018; Tchessalova and Tronson, 2019; Wang et al., 2019). Our previous studies have proved that, among CD-1 mice, gestational inflammation not only accelerates the mother’s age-related spatial learning and memory (SLM) impairment (Li X. Y. et al., 2016) but also linearly exacerbates her offspring’s age-related SLM impairment from midlife (12 months old) onward (Li X. W. et al., 2016). However, the mechanisms underlying the effect of an adverse intrauterine environment on the offspring’s age-related SLM impairment remain unknown.

Experimental evidence has indicated that numerous diseases are linked to epigenetic dysfunction, which has been viewed as an important molecular mechanism underlying the “fetal origins of adult disease” (Edwards and Myers, 2008; Bannister and Kouzarides, 2011; Brunet and Berger, 2014; Venkatesh and Workman, 2015). Our prior experiments showed that, among mice offspring with maternal LPS exposure during pregnancy, decreased acetylation of hippocampal lysine 8 (K8) of histone 4 (H4K8) and lysine 9 (K9) of histone 3 (H3K9) occurs from midlife onward, and these accelerating changes are significantly correlated with the age-related impairment of SLM (Jiang et al., 2016; Li X. W. et al., 2016; Li X. Y. et al., 2016). However, whether other abnormal post-translational modifications (PTMs, such as phosphorylation and methylation) of H3 are associated with age-related SLM impairment and are accelerated by prenatal inflammatory insult remains to be explored.

Histones (H) phosphorylation (Hp) is regulated by protein kinases and phosphatases (PPs), and it is associated with transcriptional activity (Koshibu et al., 2011). In the brain, protein kinases are known to be vital for the regulation of long-term memory and modulation of synaptic plasticity, such as that associated with long-term potentiation (Shivarama Shetty and Sajikumar, 2017). Recent studies have shown that serine/threonine protein phosphatase 1 (PP1), an important PP for phosphorylation at serine 10 (S10) of H3 (H3S10p), is known to be a suppressive molecule regarding memory formation and long-term hippocampal potentiation. Inhibiting PP1 in the amygdala can alter the H3S10p, and thereby enhance long-term fear memory (Kandel, 2001; Koshibu et al., 2011). H3p also activates the immediate-early genes, which is essential for the formation of hippocampus-dependent memory (Carter et al., 2015). A study has shown that phosphorylation at S10 and acetylation at lysine 14 (K14) of H3 (H3S10p-K14ac) work as dual H marks that are involved in behavioral changes in reaction to intense psychologically stressful events (Reul, 2014). To date, however, the functional role of the level of phosphorylated H3 in cognitive impairment has not been well elucidated. A report showed that maternal lack of n-3/n-6 polyunsaturated fatty acids during gestation significantly decreased H3S10p in the offspring hippocampi, which interfered with neurogenesis and increased the susceptibility to Alzheimer’s disease (AD; Fan et al., 2015).

In contrast to the effect of Hp on gene transcription, the consequences of H methylation (Hme) depend on the number and location of the relevant methyl groups (Bach et al., 2015). A growing number of studies have revealed that Hme is involved in memory formation. For instance, fear memory can trigger the di- or tri-methylation of K9 or K4 of H3 (H3K9me2 and H3K4me3) in the hippocampal cornu ammonis 1 (CA1; Jarome et al., 2014). Furthermore, mice that are deficient in Kmt2b (a type of H3K4 methyltransferase) perform poorly in a test of long-term memory involving object recognition and contextual fear conditioning (Kerimoglu et al., 2013). These results indicate an important role of H3 lysine methylation in long-term memory formation. However, whether H3me is involved in memory impairment has been poorly explored. JMJD2B is an H demethylase that regulates demethylation of H3K9me. A recent study found that JMJD2B-deficient mice exhibit hyperactive behaviors and deficits in working memory (Fujiwara et al., 2016). In cultured hippocampal/cortical neurons, the level of H3K9me increases significantly with age in C57BL/6 mice, and this increase is even more obvious in 3xTg-AD mice (a tri-transgenic AD model), raising the possibility that this H modification is closely associated with memory deficits (Walker et al., 2013).

Therefore, it is very interesting to hypothesize that maternal exposure to LPS could accelerate the age-related deficits of SLM in their offspring by exacerbating age-related changes in H3p and H3me levels. In the current study, therefore, we first identified whether the age-related deficits of SLM in CD-1 mice offspring (from early youth to the elderly period) could be strengthened by maternal LPS exposure during late pregnancy. Subsequently, we evaluated whether there were age-related changes in the offspring levels of H3S10p and H3K9me3 in different hippocampal subregions. Finally, we explored the correlations between worse SLM impairment and altered levels of H3S10p and H3K9me.

## Materials and Methods

### Animals and Treatments

Approximately 7- to 8-week-old CD-1 mice (males: 30–32 g, females: 26–28 g) were purchased from Beijing Vital River Laboratory Animal Technology Co. Ltd., whose foundation colonies were from Charles River Laboratories, Inc. The colony was housed in an animal room at a controlled temperature (21 ± 1°C) and humidity (55% ± 5%) with a 12-h light/dark cycle (light on: 7:00 a.m.). Food and water were provided *ad libitum*. After 2 weeks of acclimatization, male mice were paired with females (1:2) and the females were checked for vaginal plugs every morning. The presence of a vaginal plug was designated as gestational day (gd) 0. All the pregnant mice were randomly divided into three groups: higher dose-LPS group (H-LPS), lower dose-LPS group (L-LPS), and control group (CON). The mice in the LPS groups separately received intraperitoneal (i.p.) injections of LPS (50 or 25 μg/kg, *Escherichia coli* LPS, serotype 0127:B8, L3129; Sigma) at gds 15–17, based on our previous studies (Li X. W. et al., 2016; Li X. Y. et al., 2016). The CON mice received the same volume of normal saline during the same period. Mice offspring (eight males and eight females per group) were randomly selected to undergo the tasks described in Sections 2.2–2.5 (except for the mice with movement disorders, hair loss, or visible tumors) when they reached 1, 6, 12, 18, and 22 months. All animal procedures were performed in compliance with the guidelines published in the National Institutes of Health (NIH) Guide for the Care and Use of Laboratory Animals.

### Evaluation of Spatial Learning and Memory

A radial six-arm water maze (RAWM) was used to assess the SLM (Diamond et al., 1999). The apparatus was filled with water (21–22°C) and composed of a circular black tank (diameter: 100 cm, depth: 21 cm) with six swimming alleys (30.5 × 19 × 21 cm^3^) placed on a steel frame (height: 30 cm; Alzoubi et al., 2012). Each swimming alley radiated out from a central area (diameter: 40 cm). An escape platform (10 cm × 15 cm) was situated at the end of one arm, 1 cm below the surface of the water. A white curtain was hung around the apparatus from the ceiling to the ground at 75 cm from the wall, with three cardboard shapes (a circle, triangle, and square) hung at equidistance on the interior of the curtain, which acted as spatial clues. The location of the experimenter and escape platform was not changed during the entire test. 48 mice were tested in each age group (16 mice from the H-LPS, L-LPS, and control groups, respectively; half male and half female) at 8:30–10:30 every morning. The order of the mice tested daily was random. Each mouse underwent 10 consecutive days of testing, which included four consecutive identical acquisition trials (trials 1–4) and one memory retention trial (trial 5) per day. In trials 1–4, the mouse was started from one of four random alleys (not the platform alley nor its opposite alley). The mouse was allowed to find the escape platform within 60 s and stay there for 30 s. When a mouse was unable to find the platform alley (with its whole body entering the alley) or when it failed to select any alley within 10 s, it was gently guided to the beginning and an error was counted. Each consecutive acquisition trial was administered with a 30 s inter-trial interval. After the acquisition trials were completed, the mouse was placed back into its home cage and rested for 30 min. Subsequently, the mouse was released into water from the starting arm of trial 4 to carry out trial 5. The latency to find the escape platform and the number of errors from trial 1–5 were counted each day and the data were presented as means for the acquisition trials.

### Tissue Preparation

After completing the behavioral tests, the mice were anesthetized with 3% halothane in moment and decapitated. The brain tissues were then rapidly removed from the skull on ice. The tissues were bisected in the mid-sagittal position, the right hemisphere was fixed with 4% paraformaldehyde at 4°C for 3 days, and a paraffin-embedded tissue block was prepared for immunohistochemistry. Each of the paraffin-embedded tissue blocks was cut into 6-μm sections (LEICA RM 2135) and mounted on polylysine-coated slides for subsequent experiments.

### Immunohistochemistry of H3S10p and H3K9me

The streptavidin-biotin-peroxidase complex (SABC) method was used, which has been previously described in detail (Nagashima et al., 1992). Dorsal hippocampal sections were dewaxed and hydrated in graded alcohol solutions and then rinsed with tap water for 3 min. The sections were treated with periodate inactivated enzyme for 1 min (to suppress endogenous peroxidase activity) and microwaved for 20 min with citrate buffer (0.01 mol/L, pH 6.0) for antigen retrieval. Afterward, the sections were processed with 5% fetal bovine serum albumin in phosphate-buffered saline at 37°C for 10 min to minimize non-specific staining, and they were then incubated with the following primary antibodies: polyclonal rabbit anti-H3K9me (1:400; ab8898, Abcam, USA); anti-H3S10p (1:400; ab5176, Abcam, USA); or anti-total H3 (1:1,000; AH433, Beyotime, China) overnight at 4°C. On the next day, four serial procedures were performed for each section, comprising rewarming at 37°C for 40 min, incubating with a secondary antibody (biotin-labeled goat anti-rabbit) and SABC (SA1022, Wuhan Boster Bioengineering Co., Wuhan, China) for 20 min, respectively, and eventually visualizing using diaminobenzidine. The Hs are mainly located in the cellular layer, containing the dentate gyrus (DG), CA1, and CA3. The optical density (OD) values of the stained nuclei in the whole hippocampus (4 × 10) and the three subregions (the DG, CA1, and CA3, 20 × 10) were obtained with a digital camera (Nikon, Japan) equipped with an optical microscope (Olympus, Japan), and measured with Image-Pro Plus 6.0 software. In brief, the background staining was subtracted before analysis using the intensity calibration function. The intensity of the background staining and the mean value was then calculated to calibrate the incident light, which could minimize the effect of nonspecific staining. In the cellular layer of different hippocampal subregions, the areas of interest (AOI) were selected randomly to measure the OD values of immunoreactivity. The mean OD calculated using OD/AOI was viewed as the relative level of specific H. This analysis process was performed based on the double-blind principle.

### Statistical Analysis

Results were expressed as mean ± standard deviation for the data that were normally distributed. Repeated-measures analysis of variance (rm-ANOVAs) was used to analyze behavioral data, with the number of days and groups (LPS treatment group and age group) as independent variables. Immunohistochemical analysis results were tested with multivariate ANOVA to observe the main effects of maternal LPS exposure and age with sex. Fisher’s least significant difference test was performed to compare the differences among the groups. The correlations between the RAWM performance and the immunohistochemical results were analyzed with Pearson’s correlation test. *P* < 0.05 was used to represent statistical significance. The Statistical Package for Social Sciences (SPSS, version 16.0) was used to perform the data analysis.

## Results

### Radial Arm Water Maze

#### Age Effects

##### Control Groups

**Figure 1:**
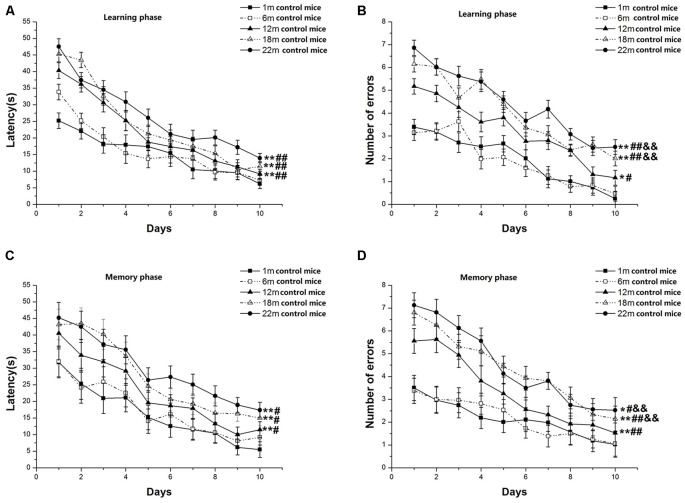
Radial six-arm water maze (RAWM) performances of control mice in different age groups. Latency **(A)** and number of errors **(B)** during the learning phase; and latency **(C)** and number of errors **(D)** during the memory phase. All values are means ± SD. *P < 0.05 and **P < 0.01 indicate significant differences compared to the 1-month-old mice; ^#^*P* < 0.05 and ^##^*P* < 0.01 indicate significant differences compared to the 6-month-old mice; ^&^*P* < 0.05 and ^&&^*P* < 0.01 indicate significant differences compared to the 12-month-old mice.

In the learning phase, latency (*F*_(9,630)_ = 87.889, *P* < 0.001; Figure 1A) and number of errors (*F*_(9,630)_ = 74.503, *P* < 0.001; Figure 1B) significantly decreased over time for all control mice combined. There was a clear effect of age on latency (*F*_(4,70)_ = 18.029, *P* < 0.001) and errors (*F*_(4,70)_ = 11.354, *P* < 0.001). *Post hoc* analysis revealed that the 22-, 18-, and 12-month-old mice had longer latency and more errors than the two younger (6- and 1-month-old) groups (*P*s < 0.05). Although there was no significant difference in the latency to reach platform between the three older groups, i.e., 22-, 18-, and 12-month-old mice (*P*s > 0.05), there was a significant increase in the number of errors in the 22- and 18-month-old mice vs. their 12-month-old counterparts (*P*s < 0.01). In the memory phase, latency (*F*_(9,630)_ = 37.103, *P* < 0.001; Figure 1C) and number of errors (*F*_(9,630)_ = 22.266, *P* < 0.001; Figure 1D) decreased over time for all mice combined. Age significantly affected latency (*F*_(4,70)_ = 4.155, *P* = 0.045) and errors (*F*_(4,70)_ = 2.846, *P* = 0.030). Further, the 22-, 18-, and 12-month-old mice had longer latency to reach the platform and more number of errors than their 6- and 1-month-old counterparts (*P*s < 0.05). There were no significant differences among 22-, 18-, and 12-month-old mice in the latency (*P*s > 0.05), but the 22- and 18-month-old mice had more errors than their 12-month-old counterparts (*P*s < 0.01). Sex and the interactions involving group × sex, group × day, sex × day, and group × sex × day had no significant effects on latency and errors (*P*s > 0.05).

##### L-LPS Groups

**Figure 2:**
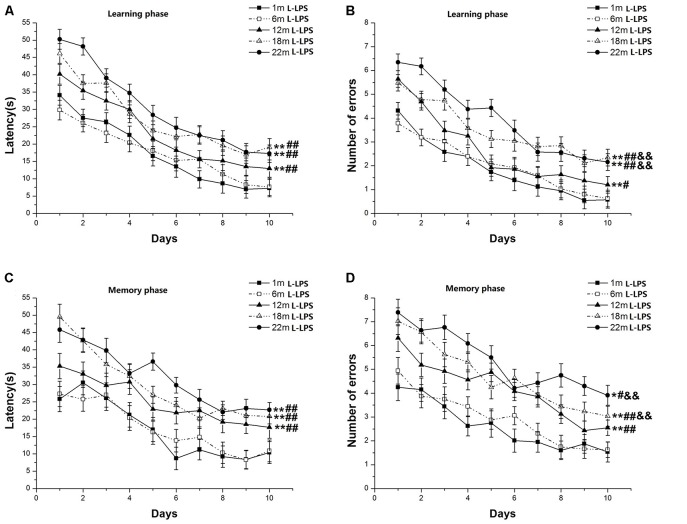
RAWM performances of L-lipopolysaccharide (LPS) mice in different age groups. Latency **(A)** and number of errors **(B)** during the learning phase; and latency **(C)** and number of errors **(D)** during the memory phase. All values are means ± SD. *P < 0.05 and **P < 0.01 indicate significant differences compared to the 1-month-old mice; ^#^*P* < 0.05 and ^##^*P* < 0.01 indicate significant differences compared to the 6-month-old mice; ^&^*P* < 0.05 and ^&&^*P* < 0.01 indicate significant differences compared to the 12-month-old mice.

Learning latency (*F*_(9,630)_ = 100.978, *P* < 0.001; Figure 2A) and number of errors (*F*_(9,630)_ = 82.825, *P* < 0.001; Figure 2B) decreased over time for all mice combined. Age significantly influenced latency (*F*_(4,70)_ = 4.641, *P* = 0.002) and errors (*F*_(4,70)_ = 5.162, *P* = 0.001). *Post hoc* analysis showed that the 22-, 18-, and 12-month-old groups had longer latencies (*P*s < 0.01) and more errors (*P*s < 0.05) than their 6- and 1-month-old counterparts. However, only the number of errors increased in the 22- and 18-month-old mice vs. their 12-month-old counterparts (*P*s < 0.01). The changed pattern of memory latency (*F*_(9,630)_ = 32.948, *P* < 0.001; Figure 2C) and errors (*F*_(9,630)_ = 37.988, *P* < 0.001; Figure 2D) was similar to the changed pattern of learning latency. Age impressively affected memory latency (*F*_(4,70)_ = 2.793, *P* = 0.033) and errors (*F*_(4,70)_ = 5.144, *P* = 0.001). Further, the 22-, 18-, and 12-month-old mice had longer latencies to reach platform and more significant increase errors than their two younger counterparts (*P*s < 0.05). Among the three older groups, the 22- and 18-month-old mice had more errors than their 12-month-old counterparts (*P*s < 0.01). Neither sex nor the interactions involving group × sex, group × day, sex × day, and group × sex × day had significant effects on the two parameters (*P*s > 0.05).

##### H-LPS Groups

**Figure 3:**
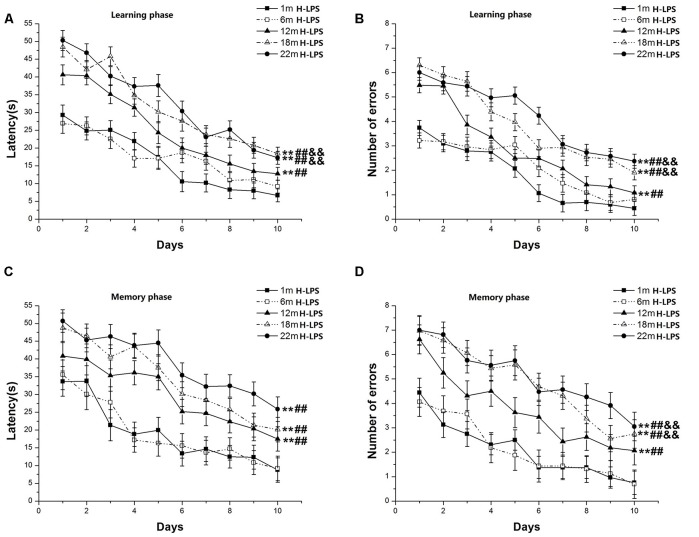
RAWM performances of H-LPS mice in different age groups. Latency **(A)** and number of errors **(B)** during the learning phase; and latency **(C)** and number of errors **(D)** during the memory phase. All values are means ± SD. *P < 0.05 and **P < 0.01 indicate significant differences compared to the 1-month-old mice; ^#^*P* < 0.05 and ^##^*P* < 0.01 indicate significant differences compared to the 6-month-old mice; ^&^*P* < 0.05 and ^&&^*P* < 0.01 indicate significant differences compared to the 12-month-old mice.

Learning latency (*F*_(9,630)_ = 81.136, *P* < 0.001; Figure 3A) and number of errors (*F*_(9,630)_ = 98.318, *P* < 0.001; Figure 3B) significantly decreased over time for all mice combined. Age had significant effects on learning latency (*F*_(4,70)_ = 7.257, *P* < 0.001) and errors (*F*_(4,70)_ = 8.370, *P* < 0.001). *Post hoc* analysis revealed that the two behavioral parameters were significant changes in the 22-, 18-, and 12-month-old mice compared to their younger counterparts (*P*s < 0.01). Furthermore, the 22- and 18-month-old mice had longer latencies to reach platform and more significant increase errors than their 12-month-old counterparts (*P*s < 0.01). In the memory phase, the latency (*F*_(9,630)_ = 33.706, *P* < 0.001; Figure 3C) and the number of errors (*F*_(9,630)_ = 39.108, *P* < 0.001; Figure 3D) also significant declined over time for all mice combined. Significant age effects existed for both the latency to reach platform (*F*_(4,70)_ = 7.715, *P* < 0.001) and the number of errors (*F*_(4,70)_ = 6.618, *P* < 0.001). *Post hoc* analysis showed that the 22-, 18-, and 12-month-old mice had more latencies and the number of errors compared to their younger counterparts (*P*s < 0.01). There were no significant differences in the latency among the 12-, 18- and 22-month-old groups (*P*s > 0.05). However, the 22- and 18-month-old mice had more errors than the 12-month-old mice (*P*s < 0.05). Sex and the interactions involving group × sex, group × day, sex × day, and group × sex × day non-significantly affected the two parameters (*P*s > 0.05).

#### LPS Treatment Effects at Different Ages

##### 1- and 6-Month-Old Mice

**Figure 4:**
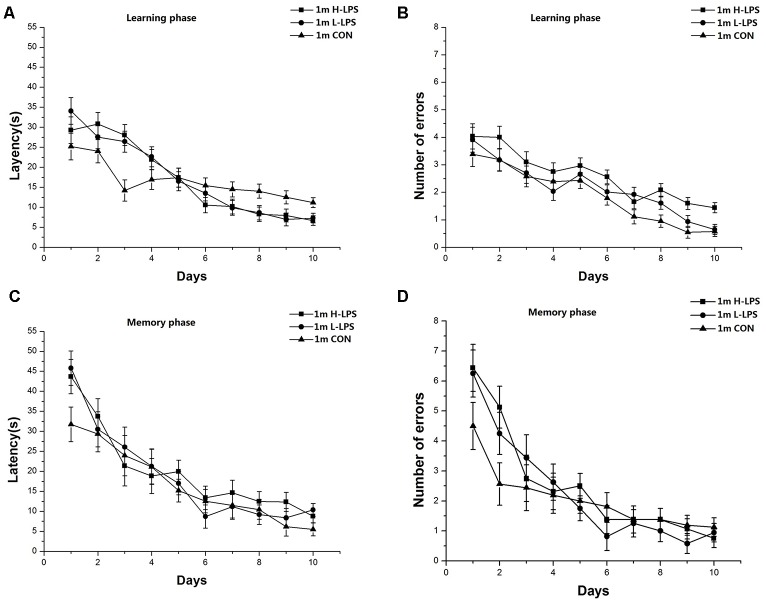
Effects of LPS treatment on the performances of 1-month-old mice. Latency **(A)** and number of errors **(B)** during the learning phase; and latency **(C)** and number of errors **(D)** during the memory phase. There were no significant treatment effects in either phase. All values are means ± SD.

**Figure 5:**
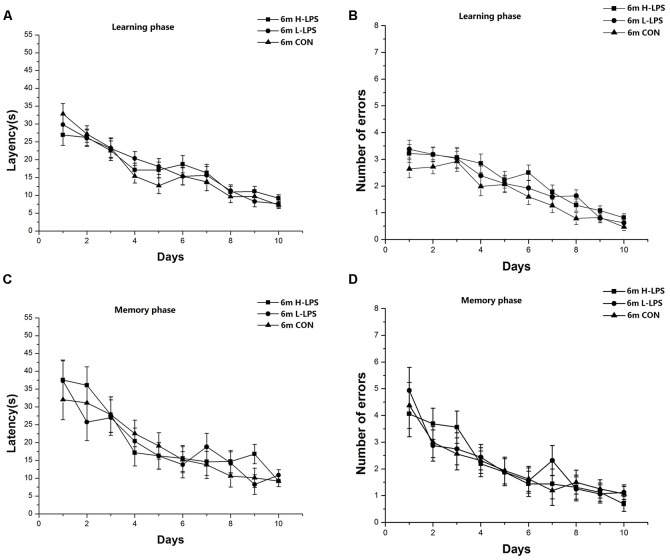
Effects of LPS treatment on the performances of 6-month-old mice. Latency **(A)** and number of errors **(B)** during the learning phase; and latency **(C)** and number of errors **(D)** during the memory phase. There were no significant treatment effects in either phase. All values are means ± SD.

**Figure 6:**
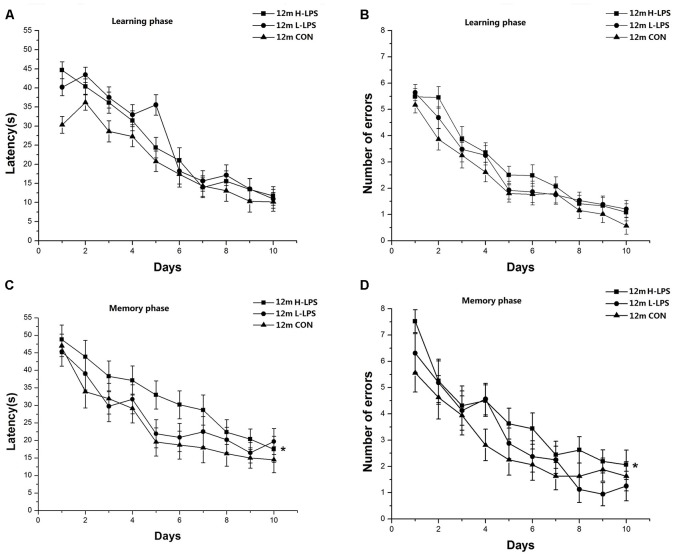
Effects of LPS treatment on the performances of 12-month-old mice. Learning latency **(A)** and number of errors **(B)**, and memory latency **(C)** and number of errors **(D)**. All values are means ± SD. There were significant treatment effects regarding the two memory phase parameters. *P < 0.05 indicates significant treatment effects compared to the same-age controls.

Latencies (1-month-old: *F*_(9,378)_ = 45.446 and 27.823, *P*s < 0.001, Figures 4A,C; 6-month-old: *F*_(9,378)_ = 35.431 and 16.708, *P*s < 0.001, Figures 5A,C) and errors (1-month-old: *F*_(9,378)_ = 64.174 and 25.235, *P*s < 0.001, Figures 4B,D; 6-month-old: *F*_(9,378)_ = 44.055 and 13.354, *P*s < 0.001, Figures 5B,D) in both the learning and memory phases declined over time for all mice combined. There were non-significant effects of LPS treatment on latencies and errors in the learning and memory phases (*P*s > 0.05). Sex and the interactions involving group × sex, group × day, sex × day, and group × sex × day also had no significant effects (*P*s > 0.05).

##### 12-Month-Old Mice

Learning latency (*F*_(9,378)_ = 80.880, *P* < 0.001; Figure 6A) and errors (*F*_(9,378)_ = 65.410, *P* < 0.001; Figure 6B) significantly decreased over time for all mice combined. The treatment effects were non-significant for latency and errors (*F*_(2,42)_ = 2.295, 1.803; *P* = 0.113, 0.177). Similarly, memory latency (*F*_(9,378)_ = 25.424, *P* < 0.001; Figure 6C) and errors (*F*_(9,378)_ = 23.328, *P* < 0.001; Figure 6D) reduced over time for all mice combined. LPS treatment significantly affected memory latency and errors (*F*_(2,42)_ = 4.984, 4.364; *P* = 0.011, 0.019). Further, the H-LPS mice had longer memory latency and more errors than the same-age CON mice (*P*s < 0.01). In both phases, sex and the interactions involving group × sex, group × day, sex × day, and group × sex × day had no significant effects on the two parameters (*P*s > 0.05).

##### 18-Month-Old Mice

**Figure 7:**
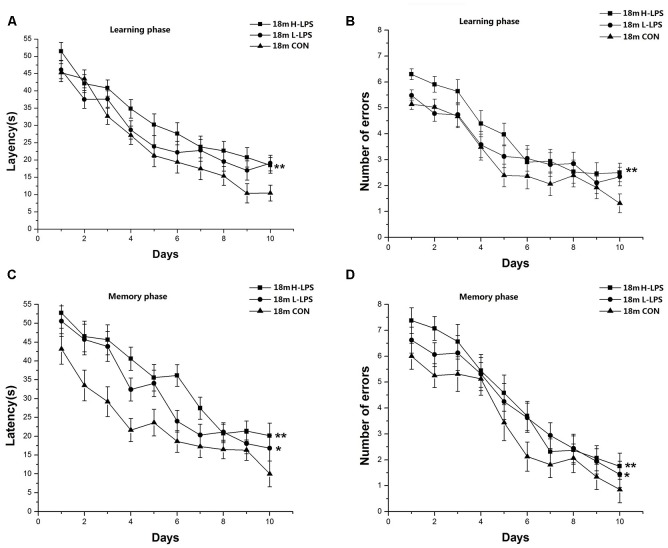
Effects of LPS treatment on the performances of 18-month-old mice. Learning latency **(A)** and number of errors **(B)**, and memory latency **(C)** and number of errors **(D)**. All values are means ± SD. There were significant treatment effects on the two learning phase parameters and two memory phase parameters. *P < 0.05 and **P < 0.01 indicate significant treatment effects compared to the same-age controls.

Learning latency (*F*_(9,378)_ = 55.566, *P* < 0.001; Figure 7A) and errors (*F*_(9,378)_ = 46.058, *P* < 0.001; Figure 7B) declined over time for all mice combined. LPS treatment significantly affected latency and errors (*F*_(2,42)_ = 5.689, 4.841; *P* = 0.007, 0.013), with longer latency and more errors in the H-LPS mice relative to the CON mice (*P*s < 0.01). The changed patterns of memory latency (*F*_(9,378)_ = 32.547, *P* < 0.001; Figure 7C) and errors (*F*_(9,378)_ = 48.842, *P* < 0.001; Figure 7D) were similar to those during learning phase. LPS treatment showed significant main effects on the memory latency and errors (*F*_(2,42)_ = 8.359, 5.984; *P* = 0.001, 0.005). Further, both the H-LPS and L-LPS mice had longer latencies and more errors than the 18-month-old CON mice (*P*s < 0.05). Sex and the interactions involving group × sex, group × day, sex × day, and group × sex × day non-significantly affected the behavioral parameters in both phases (*P*s > 0.05).

##### 22-Month-Old Mice

**Figure 8:**
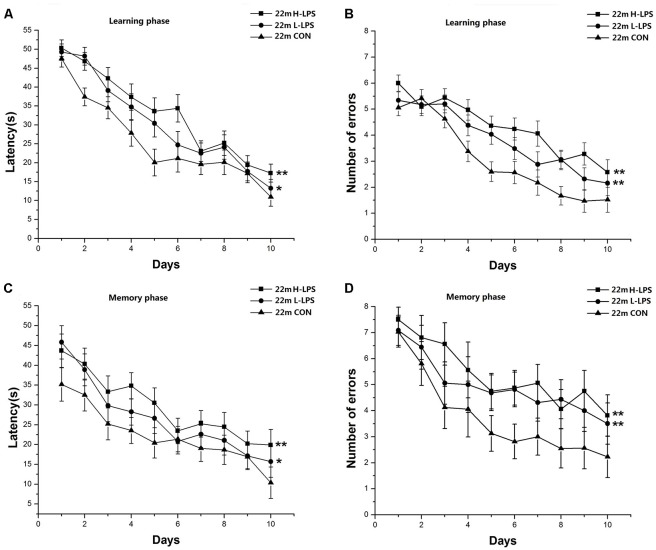
Effects of LPS treatment on the performances of 22-month-old mice. Learning latency **(A)** and number of errors **(B)**, and memory latency **(C)** and number of errors **(D)**. All values are means ± SD. There were significant treatment effects on the two learning phase parameters and two memory phase parameters. *P < 0.05 and **P < 0.01 indicate significant treatment effects compared to the same-age controls.

Learning latency (*F*_(9,378)_ = 57.393, *P* < 0.001; Figure 8A) and errors (*F*_(9,378)_ = 47.717, *P* < 0.001; Figure 8B) significantly decreased over time for all mice combined. LPS treatment had significant effects on learning latency and errors (*F*_(2,42)_ = 8.622, 10.722; *P*s < 0.01), with longer latencies and more errors in the H-LPS and L-LPS mice relative to the CON mice (*P*s < 0.05). Memory latency (*F*_(9,378)_ = 11.606, *P* < 0.001; Figure 8C) and errors (*F*_(9,378)_ = 7.082, *P* < 0.001; Figure 8D) had similar changed patterns to those in the learning phase. Latency and errors (*F*_(2,42)_ = 8.821, 8.774; *P* = 0.001, 0.001) were significantly affected by LPS treatment. Further, both the H-LPS and L-LPS mice had longer latency and more errors than the CON mice (*P*s < 0.01). In both phases, the two parameters were non-significantly affected by sex and the interactions involving group × sex, group × day, sex × day, and group × sex × day (*P*s > 0.05).

### Levels of H3K9me3, H3S10p, and Total H3 in Hippocampal Subregions

The specific immunohistochemical staining of H3K9me3, H3S10p, and total H3 were primarily observed in the cellular layer in the dorsal hippocampus, while these particular responses had a scattered distribution in the other sublayers.

#### Age Effects

**Figure 9:**
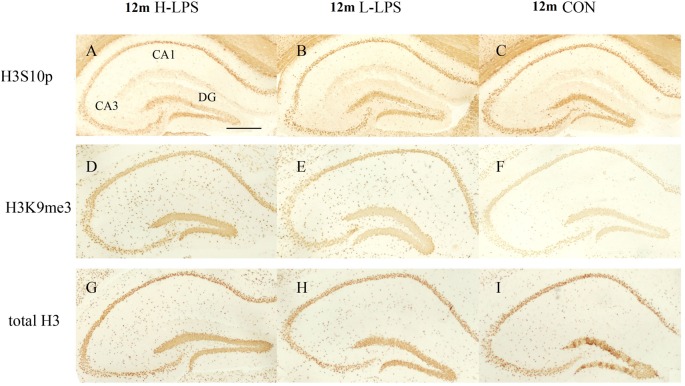
The H3S10p **(A–C)**, H3K9me3 **(D–F)**, and total H3 **(G–I)** levels in the dorsal hippocampus of 12-month-old CD-1 mice. Low-magnification images of the whole dorsal hippocampus are shown. Scale bar = 400 μm.

**Figure 10:**
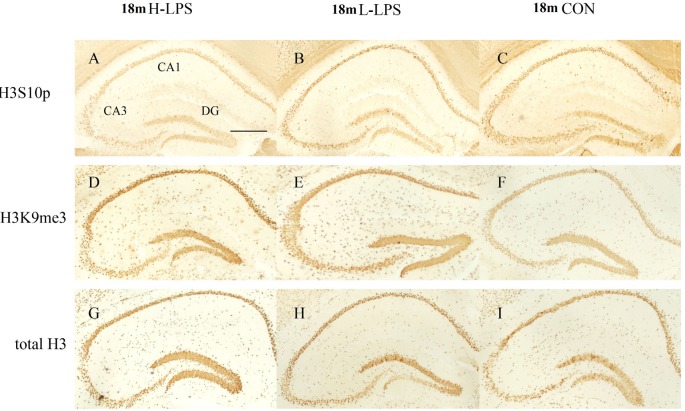
Immunoreactivities of H3S10p **(A–C)**, H3K9me3 **(D–F)**, and total H3 **(G–I)** in the dorsal hippocampus of 18-month-old mice. Low-magnification images of the whole dorsal hippocampus are shown. Scale bar = 400 μm.

**Figure 11:**
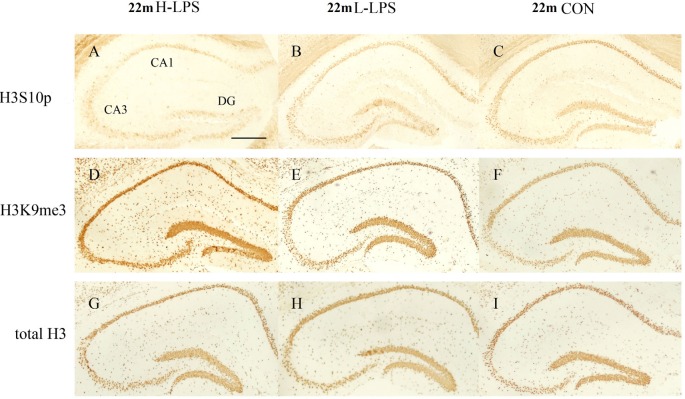
Immunoreactivities of H3S10p **(A–C)**, H3K9me3 **(D–F)**, and total H3 **(G–I)** in the dorsal hippocampus of 22-month-old mice. Low-magnification images of the whole dorsal hippocampus are shown. Scale bar = 400 μm.

**Figure 12:**
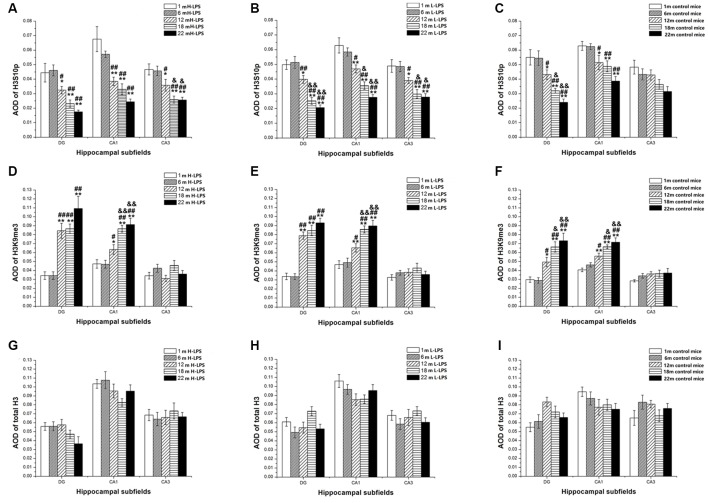
Relative levels of H3S10p **(A–C)**, H3K9me3 **(D–F)**, and total H3 **(G–I)** represented by the average optical density (AOD) of immunoreactivity in the hippocampal dentate gyrus (DG), CA1, and CA3 in different treatment groups. Panels **(A,D,G)** represent the H-LPS group; **(B,E,H)** represent the L-LPS group; and **(C,F,I)** represent the control group. All values are means ± SD. *P < 0.05 and **P < 0.01 indicate significant differences compared to the 1-month-old mice; ^#^*P* < 0.05 and ^##^*P* < 0.01 indicate significant differences compared to the 6-month-old mice; ^&^*P* < 0.05 and ^&&^*P* < 0.01 indicate significant differences compared to the 12-month-old mice.

In different months of mice, the level of H3S10p gradually decreased, and the level of H3K9me3 increased with ages (see Figures 9–11). In the CON mice, age significantly affected the levels of H3S10p and H3K9me3 in the DG (*F*_(4,75)_ = 12.970, 13.677; *P*s < 0.001) and CA1 (*F*_(4,75)_ = 8, 445, 16.791; *P*s < 0.001), but not the total H3 levels in the DG, CA1 and CA3 (*F*_(4,75)_ ≤ 1.947, *P* ≥ 0.138; see Figures 12C,F,I). Further, the DG and CA1 levels of H3S10p were lower in the 22-, 18-, and 12-month-old mice compared to the 6- and 1-month-old mice (*P*s < 0.05), as were the levels of DG H3S10p in the 22- and 18-month-old mice compared to the 12-month-old mice (*P*s < 0.05, Figure 12C). However, the three older groups had increased DG and CA1 H3K9me compared to the two younger groups (*P*s < 0.05, Figure 12F). Regarding the three older groups, the 22- and 18-month-old mice had increased DG and CA1 H3K9me compared to the 12-month-old mice (*P*s < 0.05, Figure 12F).

In the L-LPS group, age also significantly affected the levels of H3S10p and H3K9me3 in the DG (*F*_(4,75)_ = 25.590, 14.818; *P*s < 0.001) and CA1 (*F*_(4,75)_ = 21.494, 17.171; *P*s < 0.001), as well as the H3S10p levels in the CA3 (*F*_(4,75)_ = 10.274, *P* < 0.001), but not the total H3 levels in all three subregions (*F*_(4,75)_ ≤ 1.589, *P* ≥ 0.198; see Figures 12B,E,H). Further, the levels of H3S10p in the DG, CA1, and CA3 were lower in the 22-, 18-, and 12-month-old groups compared to the two younger groups (*P*s < 0.05), as did the 22- and 18-month-old mice relative to the 12-month-old mice (*P*s < 0.05, Figure 12B). Regarding H3K9me3, the 22-, 18-, and 12-month-old mice had increased DG and CA1 levels compared to the two younger groups (*P*s < 0.05), as did the 22- and 18-month-old mice relative to the 12-month-old mice in the CA1 (*P*s < 0.05, Figure 12E).

In the H-LPS group, age significantly affected the levels of H3S10p and H3K9me3 in the DG (*F*_(4, 75)_ = 11.510, 18.085; *P*s < 0.001) and CA1 (*F*_(4,75)_ = 14.964, 16.735; *P*s < 0.001), as well as the H3S10p level in the CA3 (*F*_(4,75)_ = 10.232, *P* < 0.001), but not the total H3 levels in all subregions (*F*_(4,75)_ ≤ 0.636, *P* ≥ 0.642; see Figures 12A,D,G). The 22-, 18-, and 12-month-old mice had lower H3S10p levels than the two younger groups in the DG, CA1, and CA3 (*P*s < 0.05), as did the 22- and 18-month-old mice relative to the 12-month-old mice in the CA3 (*P*s < 0.05, Figure 12A). Interestingly, the three older groups had increased DG and CA1 H3K9me3 levels relative to the two younger groups (*P*s < 0.05), as did the 22- and 18-month-old mice relative to the 12-month-old mice in the CA1 (*P*s < 0.01). The levels of H3S10p, H3K9me3, and total H3 were non-significantly affected by sex and the interactions involving group × sex in each subregion (*P*s > 0.05, Figure 12D).

#### LPS Treatment Effects

In the 6- and 1-month-old mice, the levels of H3K9me3, H3S10p, and total H3 were non-significantly different among the H-LPS, L-LPS, and CON mice (*P*s > 0.05; data not shown).

**Figure 13:**
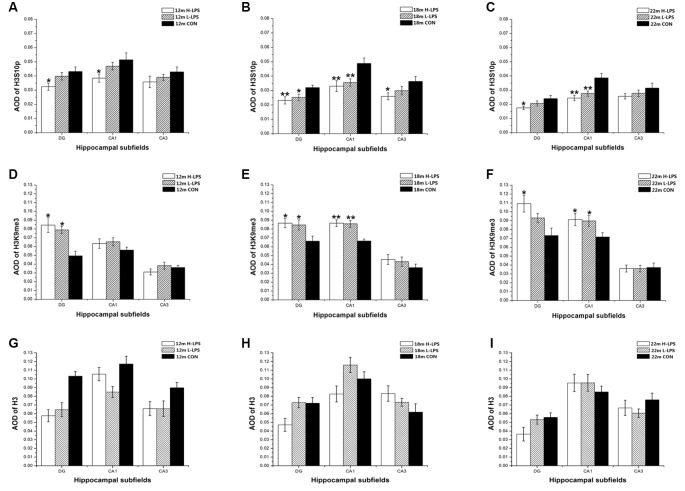
Relative levels of H3S10p **(A–C)**, H3K9me3 **(D–F)**, and total H3 **(G–I)** represented by AOD of immunoreactivity in the hippocampal DG, CA1, and CA3 in different age groups. Panels **(A,D,G)** represent 12-month-old mice; **(B,E,H)** represent 18-month-old mice; and **(C,F,I)** represent 22-month-old mice. All values are means ± SD. *P < 0.05 and **P < 0.01 indicate significant treatment effects compared to the same-age controls.

In the 12-month-old mice, LPS treatment significantly affected the levels of H3S10p in the DG (*F*_(2,45)_ = 3.649, *P* = 0.034) and CA1 (*F*_(2,45)_ = 3.279, *P* = 0.047), and H3K9me3 in the DG (*F*_(2,45)_ = 3.621, *P* = 0.035; see Figures 13A,D). More specifically, the H-LPS mice showed lower levels of H3S10p in the DG (*P* = 0.011) and CA1 (*P* = 0.015, Figure 13A) relative to the CON mice. However, the H-LPS and L-LPS mice had higher H3K9me3 levels in the DG compared to the CON mice (*P*s < 0.05, Figure 13D).

In the 18-month-old mice, LPS treatment significantly affected the levels of H3S10p and H3K9me3 in the DG (*F*_(2,45)_ = 4.628, 4.076; *P* = 0.015, 0.024) and CA1 (*F*_(2,45)_ = 6.180, 11.500; *P*s < 0.01), as well as the H3S10p level in the CA3 (*F*_(2,45)_ = 3.273, *P* = 0.047; see Figures 13B,E). Compared to the CON mice, the H-LPS and L-LPS mice showed decreased H3S10p in the DG (*P*s < 0.05) and CA1 (*P*s < 0.05), as did the H-LPS mice in the CA3 (*P* = 0.035) relative to the CON mice (Figure 13B). Regarding H3K9me, the H-LPS and L-LPS mice showed higher levels in the DG and CA1 than the CON mice (*P*s < 0.05, Figure 13E).

In the 22-month-old mice, LPS treatment significantly influenced H3S10p and H3K9me3 levels in the DG (*F*_(2,45)_ = 3.392, 3.448; *P* = 0.042, 0.040) and CA1 (*F*_(2,45)_ = 10.056, 3.318; *P* < 0.0001, = 0.045; see Figures 13C,F). Regarding H3S10p, the H-LPS mice had significantly decreased levels in the DG (*P* = 0.028) and CA1 (*P* < 0.01), and the L-LPS mice had a lower level in the CA1 (*P* = 0.002) than the CON mice (see Figure 13C). Regarding H3K9me3, the H-LPS mice had higher levels in the DG (*P* = 0.012) and CA1 (*P* = 0.031) than the CON mice, as did the L-LPS mice in the CA1 (*P* = 0.040, Figure 13F).

Regarding the 12- to 22-month-old mice, LPS treatment had no significant effects on the expression of total H3 for each subregion: DG (*F*_(2,45)_ = 1.717, 0.495, 1.514; *P*s > 0.218), CA1 (*F*_(2,45)_ = 0.475, 0.825, 0.175; *P*s > 0.457), and CA3 (*F*_(2,45)_ = 1.102, 1.093, 0.470; *P*s > 0.361; see Figures 13G–I).

Sex and the interactions involving group × sex non-significantly affected the levels of H3S10p, H3K9me3, and total H3 in each subregion (*P*s > 0.05; see Figures 12, 13).

### Correlations Between RAWM Performance and Levels of H3K9me3 and H3S10p

Regarding the 12- to 22-month-old mice, the levels of H3K9me3 and H3S10p in different hippocampal subregions were significantly associated with the learning and memory RAWM parameters, but with different patterns (Table 1).

**Table 1:** Correlations between performance of radial six-arm water maze (RAWM) and levels of H3K9me3 and H3S10p in different subregions of the hippocampus [*r*(*P*)].

Proteins	Age	Subregion	Mice	Learning phase		Memory phase	
				Latency	Number errors	Latency	Number errors
H3K9me3	12 m	CA1	All	0.342 (0.012)*	0.275 (0.059)	0.206 (0.193)	0.173 (0.239)
	18 m	DG	All	0.556 (0.000)**	0.522 (0.003)**	0.424 (0.038)*	0.491 (0.016)*
			H-LPS	0.540 (0.031)	0.457 (0.075)	0.583 (0.018)*	0.528 (0.036)*
			L-LPS	0.551 (0.027)*	0.523 (0.037)*	0.527 (0.036)*	0.447 (0.083)
		CA1	All	0.320 (0.027)*	0.304 (0.087)	0.204 (0.165)	0.190 (0.195)
	22 m	DG	All	0.106 (0.475)	0.113 (0.443)	0.318 (0.027)*	0.357 (0.013)*
			H-LPS	0.325 (0.215)	0.255 (0.341)	0.465 (0.070)	0.521 (0.038)*
		CA1	All	0.253 (0.344)	0.239 (0.373)	0.415 (0.003)**	0.402 (0.021)*
H3S10p	12 m	DG	All	−0.320 (0.026)*	−0.316 (0.029)*	−0.575 (0.000)**	−0.474 (0.001)**
			H-LPS	−0.209 (0.437)	−0.302 (0.255)	−0.593 (0.015)*	−0.618 (0.011)*
			L-LPS	−0.365 (0.164)	−0.319 (0.229)	−0.741 (0.001)**	−0.520 (0.039)**
	18 m	DG	All	−0.187 (0.203)	−0.155 (0.291)	−0.242 (0.097)	−0.330 (0.022)*
			H-LPS	−0.356 (0.176)	0.371 (0.158)	−0.451 (0.079)	−0.505 (0.046)*
		CA1	All	−0.424 (0.003)**	−0.294 (0.042)*	−0.250 (0.087)	−0.305 (0.035)*
	22 m	DG	All	−0.355 (0.013)*	−0.395 (0.005)**	−0.288 (0.047)*	−0.294 (0.042)*
			H-LPS	−0.255 (0.341)	−0.237 (0.376)	−0.435 (0.092)	−0.529 (0.035)*
		CA1	All	−0.260 (0.074)	−0.480 (0.001)**	−0.420 (0.003)**	−0.314 (0.030)*
		CA3	All	−0.330 (0.022)*	−0.293 (0.041)*	−0.235 (0.106)	−0.258 (0.077)

*Denotes significant correlation coefficients (*P < 0.05; **P < 0.01). CA, cornu ammonis; DG, dentate gyrus.

#### H3K9me3

In the 12-month-old, the only positive correlation existed in the CA1 for learning latency for all mice combined (*r* = 0.342, *P* = 0.012). In the 18-month-old, positive correlations existed in the DG regarding learning and memory latencies (*r* = 0.556, 0.424; *P* = 0.000, 0.038) and errors (*r* = 0.522, 0.491; *P* = 0.003, 0.016), and in the CA1 regarding learning latency (*r* = 0.320, *P* = 0.027) for all mice combined. When considered different treatments, DG-H3K9me3 positively correlated with memory latency and errors in the H-LPS mice (*r* = 0.583, 0.528, *P* = 0.018, 0.036), and learning and memory latencies (*r* = 0.551, 0.527; *P* = 0.027, 0.036) and learning errors (*r* = 0.523, *P* = 0.037) in the L-LPS mice. In the 22-month-old, positive correlations were noticed in the DG (*r* = 0.357, 0.318; *P* = 0.013, 0.027) and CA1 (*r* = 0.415, *P* = 0.003) regarding memory latency for all mice combined. Moreover, the H-LPS mice had a positive correlation in the DG regarding memory errors (*r* = 0.521, *P* = 0.038).

#### H3S10p

Some negative correlations with the behavioral parameters existed in different subregions in the three older groups with more correlations relative to H3K9me3. In the 12-month-old, DG-H3S10p level negatively correlated with learning and memory latencies (*r* = –0.320, –0.575; *P* = 0.026, < 0.001) and errors (*r* = –0.316, –0.474; *P* = 0.029, 0.001) for all mice. Regarding individual LPS treatment groups, DG-H3S10p negatively correlated with memory latency and errors (*r* = –0.593, –0.618; *P*s < 0.05) in the H-LPS mice, and with memory latency and errors in the L-LPS mice (*r* = –0.741, –0.520; *P*s = 0.001, 0.039). In the 18-month-old, DG-H3S10p with memory errors (*r* = –0.330; *P* = 0.022), and CA1-H3S10p with learning latency and errors (*r* = –0.424, –0.294; *P* = 0.003, 0.042) and memory errors (*r* = –0.305, *P* = 0.035) were negatively correlated for all mice. Furthermore, DG-H3S10p negatively correlated with memory errors in the H-LPS mice (*r* = –0.505, *P* = 0.046). In the 22-month-old, negative correlations existed in all three hippocampal subregions for all mice combined. In detail, negative correlations existed between DG-H3S10p with learning and memory latencies (*r* = –0.355, –0.288; *P* = 0.013, 0.047) and errors (*r* = –0.395, –0.294; *P* = 0.005, 0.042), CA1-H3S10p with learning errors (*r* = –0.480, *P* = 0.001) and memory latency and errors (*r* = –0.420, –0.314; *P* = 0.003, 0.030) and CA3-H3S10p with learning latency and errors (*r* = –0.330, –0.293; *P* = 0.022, 0.041). Furthermore, DG-H3S10p negatively correlated with memory errors (*r* = –0.529, *P* = 0.035) in the H-LPS mice.

## Discussion

### Spatial Learning and Memory Gradually Deteriorates With Aging

Age-related memory impairment is a risk factor for AD and appears to be an important factor influencing the behavioral response (Hohman et al., 2017). SLM is an essential memory component, and it is mainly dependent on the integrity of the hippocampal structure and function (McAuliffe et al., 2006; Foster, 2012). The RAWM task has been widely used to assess SLM in rodents (Paul et al., 2009), and it is highly sensitive to mild SLM impairment (Chen et al., 2004; Yang et al., 2015).

A study showed that long-term spatial memory (detected with an object-location recognition task) became impaired with aging in mice (Wimmer et al., 2012). Our prior findings suggested that SLM significantly declines with aging in SAMP8 (Chen et al., 2007) and C57BL/6 (Yang et al., 2012) mice. In the current study, CD-1 mice during late pregnancy underwent three treatments, i.e., H-LPS, L-LPS, and normal saline. The mice in each treatment group were divided into 1-, 6-, 12-, 18-, and 22-month-old groups, representing early youth, adult, midlife, old and elderly mice, respectively. The results indicate that the early youths and adults had similar performances in the RAWM, but the performance began to worsen from midlife onward in each treatment, i.e., the 12-month-old mice had more errors and longer latency in both the learning and memory phases compared to the 1- and 6-month-old mice, with even worse performances in the older and elderly mice. Our findings appear to imply that the SLM impairment of mice began in midlife, and gradually deteriorated with age until the mice were elderly, which is consistent with our previous findings (Chen et al., 2011; Li X. W. et al., 2016). A few studies showed sex differences in the decline of cognitive ability for normal aging mice, and they indicated that SLM decline began at an earlier age in females, such as 17-month C57BL/6NIA (Frick et al., 2000), 17-month Kunming (Chen et al., 2004, 2014) and 12-month CD-1 mice (Li X. W. et al., 2016). In this study, sex had no significant effects on the cognitive performance.

### Prenatal Proinflammatory Exposure Accelerates the Age-Related Impairment of Spatial Learning and Memory

Bacterial or viral infections are common during pregnancy, and they activate the maternal immune system. LPS is used widely *in vitro* and *in vivo* experimental models of neuroinflammation (Candiracci et al., 2012; MacRae et al., 2015; Anaeigoudari et al., 2016). Many researchers have used LPS-induced inflammation to explore long-term memory impairment after inflammatory stimulation (Zakaria et al., 2017). Our prior studies showed that LPS exposure during pregnancy exacerbates age-related SLM impairment in both the mothers and offspring (Chen et al., 2011; Li X. Y. et al., 2016), and the females’ damage emerged earlier than the males’ as indicated by the 12-month-old CD-1 mice whose treatment effect only occurred in the females (Li X. W. et al., 2016). In the current study, we used the same system to mimic prenatal inflammation exposure. The results showed that latencies and errors in both phases of RAWM were similar among the H-LPS, L-LPS, and CON mice aged 1 and 6 months, suggesting that inflammatory exposure within a certain dose range during the late embryonic stage did not have significant effects on SLM in youth and adulthood. This supports our previous findings (Chen et al., 2011). Regarding the 12-month-old mice, the H-LPS mice firstly exhibited damaged memory, i.e., increased memory latency and more errors, compared to the CON mice. At the age of 18 months, the H-LPS mice had impairments not only in memory but also in learning, as indicated by increased errors and latency in both phases. After entering the elderly phase (22 months old), the L-LPS mice also exhibited worse learning and memory. These results indicated that maternal LPS exposure during late gestation can result in SLM impairment in the offspring, and this treatment effect emerges from midlife (e.g., 12-month-old for CD-1 mice) onward in a dose-dependent manner, with impairments occurring earlier in memory compared with learning. This conclusion is supported by our previous findings (Chen et al., 2011; Li X. W. et al., 2016).

### Age and Prenatal Inflammatory Insult Affect Phosphorylation and Methylation of Hippocampal Histone 3

In the current study, for the first time, we observed the effects of age and prenatal inflammatory insult on H3S10p and H3K9me3 in different hippocampal subregions. Here, the semi-quantitative immunohistochemistry was utilized to assess the phosphorylation and methylation of H3 and the H3 total levels in different hippocampal subfields. We found that there was no difference on H3 total levels, and it confirmed that the change in the modified proteins was not actually a change in the core histones, but actually a change in the modification.

Regarding the age effects, the changed patterns were similar in the H-LPS, L-LPS and CON mice, i.e., hippocampal H3S10p and H3K9me3 levels in three subregions were similar at 1- and 6-month-old, but H3S10p decreased and H3K9me3 increased in the CA1 and DG at 12-month-old. At age of 18 months, the changes in H3S10p and H3K9me3 reached a peak in the severity and the number of subregions (CA3 was also affected). The peak levels seemed to be maintained until 22-month-old (with indifference compared to the 18-month-old). These results suggested that there were significant alterations in both H3 modifications from midlife (12-month-old) until the elderly for CD-1 mice, preferentially in the DG and CA1.

Regarding the effects of prenatal inflammation, the H3S10p levels decreased and the H3K9me3 levels increased from the 12-month-old onward in the LPS-treated offspring relative to the CON mice. In the 12-month-old, LPS treatment effects on the H3S10p levels significantly exhibited in the DG and CA1 of H-LPS group, and on the DG-H3K9me3 levels in both the H-LPS and L-LPS groups. In the 18-month-old, LPS treatment effects on H3S10p levels were found in all three subregions (the DG, CA1 and CA3) in the H-LPS mice, and also in the DG and CA1 in the L-LPS mice. The H3K9me3 levels increased in the DG and CA1 in both LPS groups. In the 22-month-old, LPS treatment effects on H3S10p and H3K9me3 levels were only found in the DG and CA1 of H-LPS mice, and the CA1 of L-LPS mice.

Above-mentioned findings suggest that not only in normal-aging mice but also in mice that have experienced a prenatal inflammatory insult, the inverted patterns of changes involving hippocampal H3S10p and H3K9me3 levels began in 12-month-old CD-1 mice, preferentially in the DG, then in the CA1, and lastly in the CA3, which were affected with age and severity of prenatal inflammation, and both effects had reached a peak in the 18-month-old.

### Changed Phosphorylation and Methylation of Hippocampal Histone 3 Are Associated With Age- and Prenatal Inflammation-Related Decline of Spatial Learning and Memory

We know that altering H modification or activity of H-related enzymes, such as histone deacetylases (HDACs), affects memory storage (Dash et al., 2009; Monti, 2013). But how do PTMs affect memory? An intriguing hypothesis has proposed that there is an “H code,” which regulates specific gene expression for memory formation processes. The combinatorial nature of the H code is an attractive mechanism for gene regulation, and phosphorylation and methylation of H3 may be part of an epigenetic H code for memory formation.

Our study and other studies have revealed that H acetylation is relevant to memory formation (Gräff and Tsai, 2013; Jiang et al., 2016; Li X. W. et al., 2016; Li X. Y. et al., 2016). Likely acetylation, phosphorylation is associated with transcriptional activation. Interestingly, decreasing hippocampal H3p can cause defects in spatial memory, while enhancing H3p by deleting H phosphatase PP1 can improve spatial memory in mice (Koshibu et al., 2011). H3S10p may play a vital role in activating transcription and synaptic plasticity (Jarome et al., 2014) and is related to the formation of hippocampus-dependent long-term memory (Gräff et al., 2010). The resultant H3S10p is thought to open up the chromatin structure, allowing transcription factors to bind to their responsive elements and leading to transcription of formerly silenced genes, resulting in enhance memory formation with increased H3p. Presently, there is little information on the effect of H3S10p on cognition dysfunction. A study showed that mice with defective mitogen and stress activated protein kinase 1 (MSK1), which mediated H3S10p, had impairments in fear responses and spatial memory (Chwang et al., 2007). Inhibition of PPI (reversing H3S10p) improved long-term object-recognition memory (Cohen-Armon et al., 2004; Mastroeni et al., 2010).

As for Hme, transcriptional activation is accompanied by H arginine residue methylation, while the effects of lysine residue methylation differ depending on the site of methylation. For instance, methylation of H3K4 and H3K36 activate transcription, while methylation of H3K9, H3K27, H3K79, and H4K20 repress transcription (Rivera et al., 2014). A study reported that mice deficient in the H3K4-specific H methyltransferase, Mll, display deficits in contextual fear conditioning and spatial working memory (Jakovcevski et al., 2015), suggesting that Hme are closely related to memory impairment. For the H3K9me3, a study identified that using SUV39H1 (an inhibitor of H methyl transferase) to decrease its level can improve different-type memories, including object-location memory, fear conditioning, and complex spatial environment learning tasks, and increase hippocampal brain-derived neurotrophic factor levels in aged mice (Park and Poo, 2013; Snigdha et al., 2016).

Our results indicated that for the mice combined at different ages, the levels of H3K9me3 in the CA1 in the 12-month-old, and the CA1 and DG in the 18- and 22-month-old, positively correlated with the impairment of SLM ability, and the H3S10p levels in the DG in the 12-month-old, the DG and CA1 in the 18-month-old mice, and the DG, CA1, and CA3 in the 22-month-old negatively correlated with impairment of SLM ability. These results generally indicated that the hippocampal decreased H3S10p and increased H3K9me3 levels were associated with impaired SLM ability normal-aging mice and mice experienced a prenatal inflammatory insult, beginning at 12-month-old onward in the CD-1 mice (see Table 1). Furthermore, our results also suggest that changes in H3K9me3 first occurred in the CA1 and extended to the DG with further aging (without occurring in the CA3), and changes in H3S10p first emerged in the DG and extended to the CA1 and CA3 with further aging.

For the prenatal exposure to inflammation, a positive correlation was found only between DG-H3K9me3 and latencies and/or errors in the learning and/or memory phases in the 18-month-old L-LPS and H-LPS mice, and memory errors in the 22-month-old H-LPS mice. However, a negative correlation was found between DG-H3S10p and memory errors in 12-, 18-, and 22-month-old H-LPS mice and 12-month-old L-LPS mice. These findings suggest that prenatal exposure to inflammation damages age-related memory might be more attributed to decreased H3S10p than increased H3K9me3, which firstly began in the DG.

In brief, we found, for the first time: (1) the age-related hippocampal H3S10p decrease and H3K9me3 increase began in 12-month-old CD-1 mice and continued to the elderly phase (22 months old); (2) prenatal exposure to inflammation accelerated these epigenetic changes; and (3) changes in neurobiological indicators significantly correlated with impairment of SLM abilities. Nonetheless, the study was limited by the semi-quantitative method of immunohistochemistry. This experiment ignored the distribution of the H modifications in other cognition-related brain regions. Therefore, the correlation between H3K9me3 levels and the impairment of learning and memory was not strong. In future research, we will use more accurate detection methods to clarify the mechanism of age-related memory impairment.

## Ethics Statement

This study was carried out in accordance with the recommendations of NIH Guide for the Care and Use of Laboratory Animals. The protocol was approved by the Experimental Animal Ethics Committee of Anhui Medical University.

## Author Contributions

Z-XW, WJ and X-WL carried out the experimental work. X-YL and JX participated in the data collection and interpretation. FW, G-HC, Z-XW and LC participated in the design and coordination of experimental work. Z-XW and LC participated in the analysis of data and preparation of the manuscript. All authors read and approved the final manuscript.

## Conflict of Interest Statement

The authors declare that the research was conducted in the absence of any commercial or financial relationships that could be construed as a potential conflict of interest.
